# A Study of Efficacy and Safety of Ashwagandha (Withania somnifera) Lotion on Facial Skin in Photoaged Healthy Adults

**DOI:** 10.7759/cureus.36168

**Published:** 2023-03-15

**Authors:** Keerthi Narra, Santosh K Naik, Anjali S Ghatge

**Affiliations:** 1 Dermatology, Skintis Clinic, Hyderabad, IND; 2 Pharmacology, Kamineni Academy of Medical Sciences and Research Centre, Hyderabad, IND; 3 Aesthetic Dermatology, Apollo Clinic, Mumbai, IND

**Keywords:** photoageing, ashwagandha, skin hydration, sf-12, transepidermal water loss

## Abstract

Background

Facial skin has an essential cosmetic function in both men and women, and photoaged skin can affect the quality of life in healthy people. Ashwagandha (*Withania somnifera*) which is also called Indian ginseng has adaptogenic properties and is used in traditional Indian medicine to maintain balance, energize, and rejuvenate.

Objective

This randomized, double-blind, and placebo-controlled study assessed the efficacy and safety of topical application of lotion containing 8% standardized Ashwagandha root extract on improvement of skin parameters in the photoaged facial skin of healthy subjects.

Methods

Fifty-six healthy men and women aged between 18 and 60 years with Fitzpatrick phototype III-VI skin grade were randomized to receive the topical application (lotion on facial skin) of either Ashwagandha 8% (AG, n=28), or an identical placebo (PL, n=28) for 60 days. The primary outcome was the change from baseline on day 60 in the scores for global physician assessment scoring for the five dermatological signs (skin wrinkles, pores, hydration/moisture, skin brightness/tone, and pigmentation) on facial skin. Secondary outcomes were changes from baseline in the transepidermal water loss (TEWL), melanin index, hydration, and skin elasticity (R2 ratio). Another efficacy outcome was quality of life using the health-specific Short Form Health Survey-12 (SF-12). Safety was assessed using local reactions and adverse events. Three (1 AG, 2 PL) patients were lost to follow-up and per-protocol (PP) data included 53 patients (27 AG, 26 PL). For measurement data, repeated measures analysis of variance (ANOVA) was used to assess treatment effect at different time periods in the PP dataset (n=53). Two groups were compared for differences using a t-test for continuous data or a Mann-Whitney ‘U’ test for ordinal data. Adverse events were compared between two groups using the chi-square test.

Results

Greater reduction (p<0.0001) in total physician assessment scores from baseline to day 60 was observed with AG (-74.69%) compared to PL (-48.68%). There was a greater improvement in TEWL, skin hydration, and skin elasticity (R2 ratio) with AG as compared to placebo (p<0.0001). However, the change in melanin index was similar in the two groups at the end of day 60 (p=0.969). The percentage increase in melanin index from baseline to day 60 in the PP dataset was by -2.82% with AG and -1.78% with PL, whereas the percentage reduction in TEWL from baseline to day 60 in the PP dataset was by -15.12% with AG and -8.34% with PL. Similarly, greater percentage improvements were seen with AG as compared to PL for skin hydration (20.66% with AG and 9.5% with PL) and elasticity was assessed by the R2 ratio (16.34% with AG and 3.73% with PL). Adverse events were comparable in the two groups.

Conclusions

Topical application of a lotion containing Ashwagandha standardized root extract improves the skin condition and quality of life in photoaged healthy individuals. Further studies with different skin types and standard comparators are warranted to substantiate these claims of benefit.

## Introduction

Skin covers the body, and its main function is to safeguard the internal organs from the external environment [1]. Facial skin has an essential cosmetic function in both men and women. Skin aging is commonly manifested as wrinkles, reduced tone, sagging, and dry skin which can significantly impact self-esteem and social relations. As reported by Mukherjee et al., skin aging is influenced by several factors, including genetics, environmental exposure (ultraviolet rays, xenobiotics, and mechanical stress), hormonal changes, and metabolic processes (generation of reactive chemical compounds such as activated oxygen species, sugars, and aldehydes) [2]. Although multiple factors contribute towards the alterations of skin structure, function, and appearance, exposure to solar ultraviolet (UV) radiation is one of the prominent factors causing skin aging [3].

Skin is the largest organ of the human body, and maintaining a healthy skin condition directly promotes a healthy body [4]. Photoaging is the result of changes or damage in the skin due to exposure to ultraviolet radiation from sunlight. Photoaged skin has a different dermal extracellular matrix morphology with solar elastosis (deposition of dystrophic elastic fibers in the dermis) being a prominent histological feature [5]. UV exposure enhances the aging of intrinsically aged skin causing premature aging. Clinically, the skin becomes coarse, with thickening of the epidermis (hyperplasia) followed by atrophy, laxity, sallowness (dark discoloration) with wrinkles, irregular hyperpigmentation, lentigines, and telangiectasias [6]. The skin pores in photoaged skin are larger and tend to develop Favre-Racouchot’s syndrome (nodular elastosis with cysts and comedones). This is associated with an increase in the development of benign neoplasms (seborrheic keratosis, fibroma, acrochordon, and ruby spots), “premalignant” lesions (actinic keratosis, lentigo maligna), and malignant lesions (basal and squamous cell carcinomas and malignant melanomas) on chronically exposed skin found in the face, hands, and neck regions [7,8]. Inflammatory skin conditions, such as allergic contact dermatitis, psoriasis, and atopic dermatitis, are associated with the function of keratinocytes and cytokines [9].

Traditional medicinal systems have used herbal therapies to treat hair and skin conditions since ancient times, and many studies have discovered anti-inflammatory and wound-healing activities in substances extracted from plants [10-12]. Ashwagandha (*Withania somnifera*, {WS} family Solanaceae) is one of the most important herbs of the Indian system of medicine Ayurveda and is used for its multiple health benefits [13]. WS has adaptogenic properties and is used in traditional Indian medicine to maintain balance, energize, and rejuvenate [14,15]. WS is one of the extensively prescribed botanicals in Ayurveda practice for its multimodal effects [16]. The diverse pharmacological activities including immunomodulatory, anti-inflammatory, antioxidant, anti-stress, anti-hypertensive, and anti-diabetic along with organ-protective effects have been studied extensively [13]. The chemical characteristics of different extracts used in WS formulations are well documented. WS extracts contain different phytochemical agents like withanolides which provide strong therapeutic benefits [17]. Ashwagandha used in the form of a paste of boiled roots is reported to have wound-healing abilities in Ayurveda literature [18]. This suggests that one or more components of WS have active physiological effects on the skin. Studies on topical treatment with WS have confirmed their chemopreventive effects on skin cancer and their melanin regulatory effects [19,20].

This placebo-controlled study assessed the efficacy and safety of the topical application of Ashwagandha root extract on the improvement of skin parameters in the photoaged skin of healthy subjects.

## Materials and methods

Study design and setting

This prospective, randomized, placebo-controlled, comparative study was conducted at Skintis Clinic, Hyderabad, India. The study was conducted in accordance with the Helsinki Declaration (1989 amendment) and the study protocol was approved by the Deccan Independent Ethics Committee, Hyderabad, India (dated October 31, 2022). The study was registered with the clinical trials registry of India with #CTRI/2022/11/047537. The Consolidated Standards of Reporting Trials (CONSORT) guidelines for designing and reporting controlled trials were followed in conducting and reporting this study. Written informed consent was obtained from all participants prior to the enrolment. Each participant was explained in detail about the study objective and the expected outcome before obtaining consent.

Study participants

Healthy men and women aged between 18 and 60 years (both inclusive) visiting the study site between October and December 2022 were screened for study eligibility based on the study eligibility criteria. Participants with Fitzpatrick phototype III-VI skin grade who visited the clinic for improvement of facial skin condition and agreed not to use any oral medication or topical application (prescription and over the counter) other than study medication, including vitamins and minerals, during the study were enrolled and randomized. Participants agreed to perform study-related skin assessments. Participants having any clinically significant medical history, medical findings including rosacea, eczema, psoriasis, and atopic dermatitis, or an ongoing medical or psychiatric condition were not enrolled. Those with a history of hypersensitivity reactions who received any medicated acne treatment within the last six months, or with an active skin disease on the face were excluded. Participants with a history of dysplastic nevi or melanoma on the face, or having moles, cysts, tattoos, scars, or irritated skin on the face were also excluded. Also, those with any dermatological condition and those with any esthetic, cosmetic, or dermatological treatment in the face area within the last 30 days were not included. Participants on systemic therapy with immunosuppressive drugs (e.g., corticosteroids) and/or anti-histamines within seven days prior to the start of the study and/or throughout the entire course of the study were not included. Those who received any systemic anti-microbials within 30 days prior were excluded. Participants who were outdoor workers with a possibility of exposure to sun and those who participated in a clinical study during the preceding 180 days were excluded from participation.

Blinding and randomization

Eligible participants were randomly assigned to Ashwagandha (AG) group or placebo (PL) group in a 1:1 randomization ratio. A computer-generated (Rando version 1.2 for Windows) predetermined randomization chart was prepared. Both study and control treatment formulations were manufactured and packed in identical containers and labeled uniformly to ensure blinding. Randomization codes were provided to investigator in separate sealed envelopes for each study participant to ensure concealment. An independent investigator who was blinded for treatment received by the participants assessed the study outcomes.

Interventions

Participants in the study (AG) group received lotion (Punarved Bioceutical Private Ltd, Mumbai, India) containing 8% standardized Ashwagandha root extract (KSM-66®: Ixoreal Biomed, Hyderabad, India), whereas those in the placebo (PL) group received identical lotion (Punarved Bioceutical Private Ltd, Mumbai, India) without Ashwagandha. The root extract was standardized to contain 5% withanolides, the main active ingredient in Ashwagandha roots. Both lotions were dispensed in identical containers and labels. Participants were instructed to use a standard cleanser (simple skin moisturizing facial wash) twice daily (morning and evening/night) and apply the study lotion immediately after cleansing. Participants were instructed to apply 1 mL of skin lotion on the face until it was well absorbed. A duration of minimum of 8 hours was maintained between the two applications. The lotion was used for a period of 60 days at home, and the participants maintained a diary to assess medication compliance.

Study outcomes

The primary outcome was the change from baseline in the total and individual scores for global physician assessment scoring for the five dermatological signs (skin wrinkles, pores, hydration/moisture, skin brightness/tone, and pigmentation) on facial skin. Secondary outcomes were changes from baseline in the transepidermal water loss (TEWL), melanin index, hydration, and sebum content in the facial skin. Other efficacy outcomes were changes from baseline in the patient-reported scores for quality of life using the Short Form Health Survey-12 (SF-12) Survey questionnaire [21]. Safety was assessed using the number and proportion of treatment-emergent adverse events (TEAEs) over a 60-day treatment period.

Skin assessments by a dermatologist

A blinded dermatologist conducted the overall skin condition of the face at baseline and after day 60 using digital images of facial skin. Photographic images were captured utilizing Canfield VISIA-CR digital imaging system (Canfield Scientific Inc, Parsippany-Troy Hills, NJ). The parameters for global physician assessment of skin were skin wrinkles, pores, hydration/moisture, skin brightness/tone, and pigmentation. These signs were scored using a six‐point Likert scale of 0=none, 1=minimal, 2=mild, 3=moderate, 4=moderately severe, and 5=severe. A higher score indicates a poor skin condition. The total scores were computed as the sum of individual scores (range: 0-25) and presented as total global physician assessment scores.

Skin assessments by Cutometer® Dual MPA 580

Skin assessments were done at baseline and after 60 days (at end of the study) by a dermatologist in a double-blinded fashion. Subjects were required to rinse their face thoroughly with a neutral lotion and acclimatized them to the ambient environment for at least 15 minutes before measurements on both upper cheeks in well-defined measurement locations. Cutometer® Dual MPA 580 (Courage+Khazaka electronic GmbH, Cologne, Germany) was used for skin assessments. Skin elasticity was measured using the R2 ratio parameter (Cutometer®). R2 is the ratio of measurements with the Cutometer at complete relaxation and penetration immediately after the suction procedure. Mexameter MX18® (Courage+Khazaka electronic GmbH, Cologne, Germany) was used to determine skin color using melanin index, and the moisture content of the skin was measured by Corneometer® (Courage+Khazaka electronic GmbH, Cologne, Germany). Mexameter is a spectrometer based on light reflection and absorption. The probe emits three wavelengths of light, chosen to correspond to the different absorption rates of melanin and hemoglobin. This light emitted by the probe is reflected by the skin and the receiver in the probe measures this reflected light. It is only the diffuse and scattered light that is measured. The results are shown in 1 second as index numbers between 0 and 999. A higher value indicates higher melanin content of the skin. The Corneometer® measures the capacitance of the skin and estimates the water content of the stratum corneum of skin at a depth of 10-20 μm. A higher reading indicates increased moisture and an improved barrier function of the skin. Transepidermal water loss (TEWL) measures the rate at which water is lost by the skin and the reading obtained is then used to estimate the water retention ability of the skin thus reflecting on the permeability and efficiency of the skin barrier function [22]. It is commonly used as a diagnostic tool for skin disorders and to study the effect of substances on the skin [23]. TEWL (g/h m^2^) was assessed by Tewameter TM300® (Courage+Khazaka electronic GmbH, Cologne, Germany).

Short Form Health Survey-12

The SF-12 is a self-reported outcome reported by patients to assess the impact of health on an individual's everyday life. The SF-12 assesses the mental domain score (MCS-12) and physical domain score (PCS-12) of the individual on a 12-item questionnaire. Scores are reported for MCS-12, PCS-12, and total SF-12. The total score ranges from 12 to 47, with a higher score indicating good quality of life (QoL). Study participants completed the English version of the survey at baseline and on day 60.

Safety outcomes and compliance

The safety was assessed based on the number and proportion of treatment-emergent adverse events (TEAEs) and treatment-emergent serious adverse events (TESAE) during the study period. Compliance was assessed based on the participant diary and the returned content of the lotion, and the patient was considered compliant if more than 80% of the medication was consumed as per the protocol.

Sample size

The study sample size was determined based on the improvement (mean % reduction) in skin wrinkles score by 6.2% (1.6%) from baseline to week 4 with pomegranate extract [24]. The mean (SD) reduction in wrinkles score was 1.0% (1.4%). Group sample sizes of four in each of the two groups provide 90% power to detect a difference in wrinkles scores by 5.2 (assuming a null hypothesis that both group means are 6.2 and the alternative hypothesis that the mean of group 2 {PL} is 1.0 with known group standard deviations of 1.6 and 1.4), at a significance level (alpha) of 0.0500 with a two-sided two-sample t-test. However, we planned to include 28 participants in each of the two treatment groups.

Statistical methods

All statistical analyses were done with a windows-based program MedCalc® statistical software version 20.018 (MedCalc Software Ltd, Ostend, Belgium; https://www.medcalc.org; 2023). Efficacy analysis was performed on the per-protocol (PP) data (n=53), whereas safety analysis was performed on the intent-to-treat (ITT) data (n=56). Summary statistics for all the parameters were performed and the results were presented as means with standard deviation (SD) and 95% confidence interval (CI) for continuous data, whereas categorical data are presented as counts with percentage (%). Categorical and nominal data were analyzed for differences between the two groups using the chi-square test (Fisher’s exact test). For measurement data, between-group comparisons were analyzed using an independent sample t-test. Repeat measures analysis of variance (ANOVA) using the general linear model was used for assessment of treatment effect at different time periods, with post hoc independent sample t-test for between-group comparisons. For ordinal data, between-group comparisons were analyzed using the Mann-Whitney U test. Two-sided tests were used for analyses at alpha 0.05 (95% confidence levels).

## Results

Demography and baseline data

**Figure 1:**
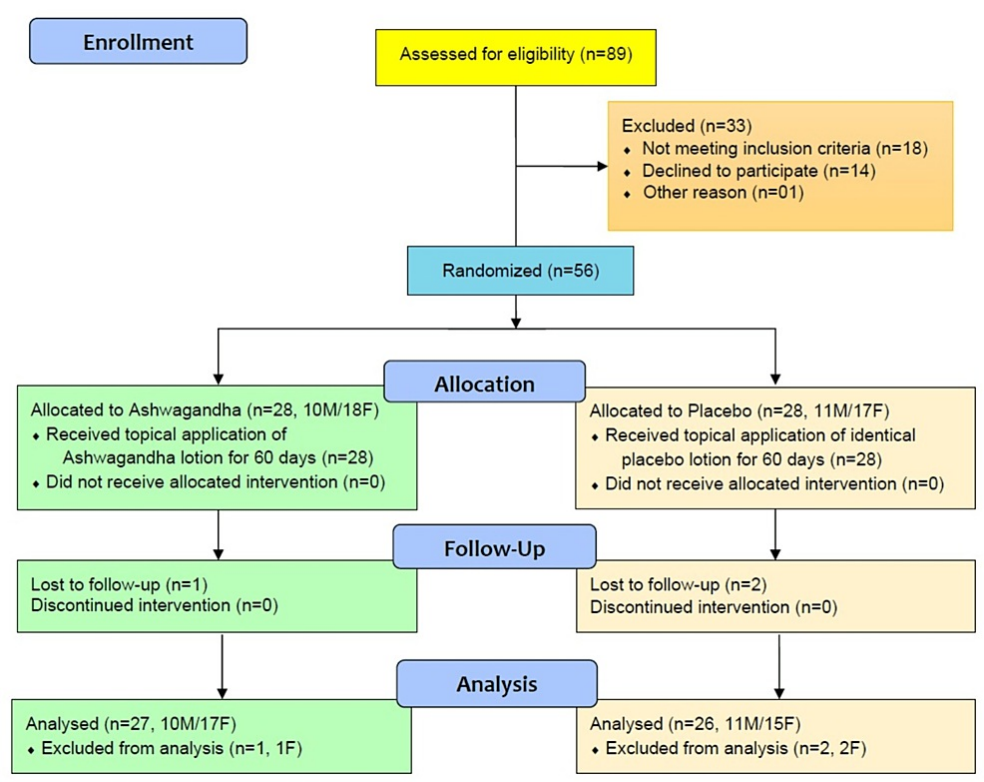
CONSORT flow diagram of the clinical study on efficacy and safety measurement of Ashwagandha lotion on facial skin in photoaged healthy adults. CONSORT: Consolidated Standards of Reporting Trials; M: male; F: female

Eighty-nine prospective patients were screened and assessed for eligibility. About 33 participants failed study eligibility, and 56 participants (21 male, 35 female) were randomized to receive either Ashwagandha lotion (n=28) or placebo (n=28) (CONSORT flow chart, Figure 1).

One participant from the AG group and two from the PL group did not visit for follow-up assessments and were not included in efficacy analysis. The final efficacy analyses were done on the per-protocol (PP) dataset of 53 (27 AG and 26 in the PL group) participants. Safety analyses were done on all 56 participants who received at least one application of lotion in the intent-to-treat (ITT) dataset. Patients in the PP dataset were between the ages of 18 and 40 years.

The two groups (AG and PL) were similar with respect to the demographic characteristics (Table 1). Two patients each had hypertension in AG and PL groups, whereas two patients had diabetes in the AG group and one in the PL group. One patient in the PL group had asthma. All patients continued their medication for concomitant illness throughout the study. The baseline assessments for physician global assessment scores, skin parameters by cutometer, and SF-12 scores were similar in the two groups (Table 2).

**Table 1:** Demography and baseline vital parameters in patients randomized (n=56). *Chi-square test (between group comparisons). **Mean (SD). ***Independent sample t-test (between group comparisons). BMI: body mass index; SD: standard deviation; CI: confidence interval

Variables		Ashwagandha (n=28) (%)	Placebo (n=28) (%)	Mean difference (95% CI)	Test	p-Value
Gender	Male	10 (35.7%)	11 (39.3%)	-	0.076*	0.783
	Female	18 (64.3%)	17 (60.7%)	-	-	-
Age (years)		30.54 (7.66)**	31.50 (6.34)**	-0.96 (-4.73 to 2.80)	-0.513***	0.610
BMI (kg/ m^2^)		30.24 (4.68)**	29.40 (3.66)**	0.83 (-1.41 to 3.08)	0.744***	0.460

**Table 2:** Baseline scores in two groups for the randomized patients (n=56). *Independent sample t-test for between-group comparisons. **Mann-Whitney U test for between-group comparisons. QoL: quality of life; PCS-12: physical domain of SF-12 QoL; MCS-12: mental domain of SF-12 QoL; SF-12: Short Form Health Survey-12; TEWL: transepidermal water loss; SD: standard deviation; CI: confidence interval Total physician assessment score is the sum of scores for individual signs (wrinkles, pores, hydration/moisture, brightness/tone, and pigmentation).

Parameter		Ashwagandha (n=28)	Placebo (n=28)	Mean difference (95% CI)	Test	p-Value
		Mean (SD)	Mean (SD)			
QoL scores	PCS-12 score	11.82 (1.98)	11.75 (2.17)	0.07 (-1.04 to 1.19)	0.129*	0.898
	MCS-12 score	17.46 (1.14)	17.18 (1.49)	0.29 (-0.43 to 1.00)	0.806*	0.424
	SF-12 total score	29.29 (2.57)	28.93 (3.03)	0.36 (-1.15 to 1.86)	0.476*	0.636
Cutometer parameters	TEWL (g/h m^2^)	22.73 (04.72)	22.57 (04.27)	0.16 (-2.25 to 2.57)	0.134*	0.894
	Melanin index (AU)	381.85 (79.23)	377.21 (73.12)	4.64 (-36.21 to 45.49)	0.228*	0.821
	Hydration (CU)	24.65 (04.01)	24.79 (05.00)	-0.15 (-2.57 to 2.28)	-0.121*	0.904
	R2 ratio	0.63 (0.13)	0.62 (0.14)	0.004 (-0.07 to 0.08)	0.124*	0.902
Physician assessment scores	Skin wrinkles	1.61 (0.79)	1.57 (0.74)	0.036 (-0.37 to 0.44)	0.175**	0.862
	Pores	2.04 (0.74)	2.07 (0.77)	-0.036 (-0.44 to 0.37)	-0.177**	0.860
	Hydration/moisture	1.64 (0.99)	1.68 (1.02)	-0.036 (-0.57 to 0.50)	-0.133**	0.895
	Brightness/tone	1.82 (0.72)	1.79 (0.69)	0.036 (-0.34 to 0.41)	0.190**	0.850
	Pigmentation	2.11 (0.69)	2.07 (0.66)	0.036 (-0.32 to 0.39)	0.198**	0.844
	Total physician assessment score	10.11 (2.47)	10.07 (2.43)	0.036 (-1.28 to 1.35)	0.055**	0.957

Physician’s global assessment

**Figure 2:**
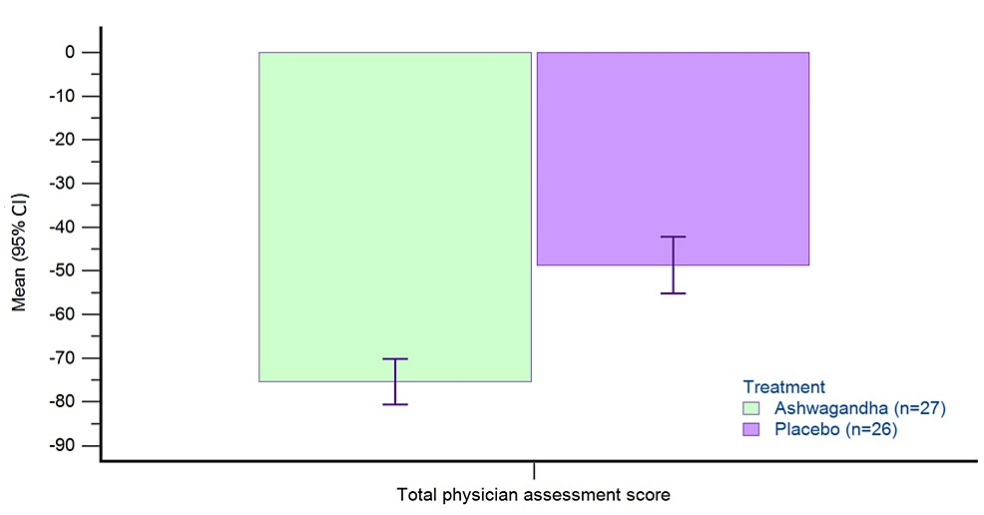
Percent change from baseline in total physician’s assessment score on day 60 in PP dataset. Total physician assessment score is the sum of scores for individual signs scores (wrinkles, pores, hydration/moisture, brightness/tone, and pigmentation). Higher scores indicate a poor skin condition. PP: per-protocol; CI: confidence interval

Table 3 presents the baseline and change from baseline data of physician assessment scores for individual signs and total scores in the per-protocol dataset (n=53). There is a greater reduction in all scores (individual and total) with AG as compared to placebo (p<0.05). The percent reduction in total physician assessment scores from baseline to day 60 in the PP dataset was -74.69% with AG and -48.68% with PL (Figure 2).

**Table 3:** Physician assessment scores in PP dataset (n=53). *Mann-Whitney U test (between-group comparisons). Total physician assessment score is the sum of scores for individual signs scores (wrinkles, pores, hydration/moisture, brightness/tone, and pigmentation). Higher scores indicate poor skin conditions. SD: standard deviation; CI: confidence interval; PP: per-protocol

Parameter	Treatment	N	Baseline	Change from baseline on day 60		Between-group comparisons		
			Mean (SD)	Mean (SD)	Mean difference (95% CI)	Test*	p-Value	Effect size -Cohen's “d” (95% CI)
Skin wrinkles score	Ashwagandha	27	1.59 (0.80)	-0.89 (0.70)	-0.50 (-0.86 to -0.15)	-2.872	0.006	-0.789 (-1.345 to -0.226)
	Placebo	26	1.58 (0.76)	-0.38 (0.57)				
Pores score	Ashwagandha	27	2.00 (0.73)	-1.33 (0.78)	-0.45 (-0.83 to -0.07)	-2.349	0.023	-0.645 (-1.195 to -0.090)
	Placebo	26	2.08 (0.74)	-0.88 (0.59)				
Hydration/moisture score	Ashwagandha	27	1.63 (1.01)	-1.41 (0.97)	-0.64 (-1.12 to -0.15)	-2.651	0.011	-0.729 (-1.282 to -0.168)
	Placebo	26	1.69 (0.97)	-0.77 (0.76)				
Brightness/tone score	Ashwagandha	27	1.78 (0.70)	-1.37 (0.74)	-0.52 (-0.88 to -0.16)	-2.926	0.005	-0.804 (-1.361 to -0.240)
	Placebo	26	1.77 (0.71)	-0.85 (0.54)				
Pigmentation score	Ashwagandha	27	2.11 (0.70)	-1.70 (0.67)	-0.47 (-0.82 to -0.13)	-2.732	0.009	-0.751 (-1.305 to -0.189)
	Placebo	26	2.04 (0.66)	-1.23 (0.59)				
Total physician assessment score	Ashwagandha	27	10.04 (2.49)	-7.63 (2.42)	-2.75 (-4.02 to -1.47)	-4.334	<0.0001	-1.19 (-1.77 to -0.60)
	Placebo	26	9.92 (2.45)	-4.88 (2.18)				

Skin assessments by Cutometer®

Table 4 presents the baseline values and change from baseline values for different skin parameters assessed by Cutometer® in the per-protocol dataset. There was a greater improvement in transepidermal water loss (TEWL), skin hydration (moisture), and skin elasticity (R2 ratio) with AG as compared to placebo (p<0.0001). However, the change in melanin index was similar in the two groups at the end of day 60 (p=0.969).

**Table 4:** Cutometer parameters in PP dataset (n=53). *Post hoc independent sample t-test (between-group comparisons). P value <0.05 (except for melanin index) for time effect in repeat measures ANOVA. TEWL: transepidermal water loss; R2 ratio: skin elasticity; SD: standard deviation; CI: confidence interval

Parameter	Treatment	N	Baseline	Change from baseline on day 60		Between-group comparisons		
			Mean (SD)	Mean (SD)	Mean difference (95% CI)	Test*	p-Value	Effect size - Cohen's “d” (95% CI)
TEWL (g/h m^2^)	Ashwagandha	27	22.90 (04.71)	-3.41 (0.82)	-1.44 (-1.91 to -0.97)	-6.137	<0.0001	-1.686 (-2.310 to -1.050)
	Placebo	26	22.55 (04.41)	-1.97 (0.89)				
Melanin index (AU)	Ashwagandha	27	384.79 (79.17)	168.38 (185.75)	-1.97 (-104.08 to 100.13)	-0.039	0.969	-0.011 (-0.549 to 0.528)
	Placebo	26	376.82 (75.52)	170.35 (184.43)				
Hydration (CU)	Ashwagandha	27	24.89 (03.86)	5.28 (1.27)	2.87 (2.17 to 3.56)	8.306	<0.0001	2.28 (1.58 to 2.97)
	Placebo	26	25.07 (04.78)	2.41 (1.24)				
R2 ratio	Ashwagandha	27	0.63 (0.13)	0.10 (0.01)	0.08 (0.07 to 0.08)	22.045	<0.0001	6.057 (4.76 to 7.34)

**Figure 3:**
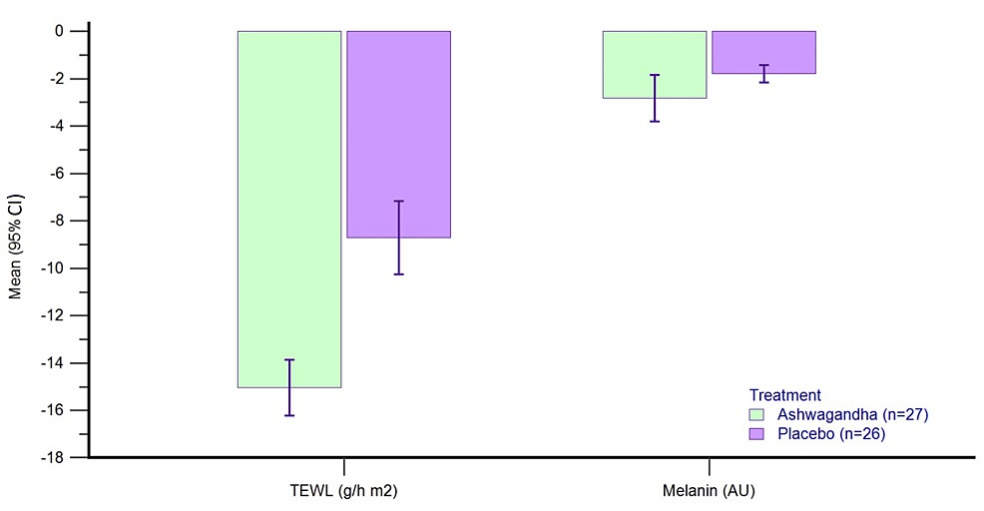
Percent change from baseline in TEWL and Melanin index on day 60 in PP dataset. TEWL: transepidermal water loss; PP: per-protocol; CI: confidence interval

The percent decrease in melanin index from baseline to day 60 in the PP dataset was by -2.82% with AG and -1.78% with PL, whereas the percent reduction in TEWL from baseline to day 60 in the PP dataset was by -15.12% with AG and -8.34% with PL (Figure 3).

**Figure 4:**
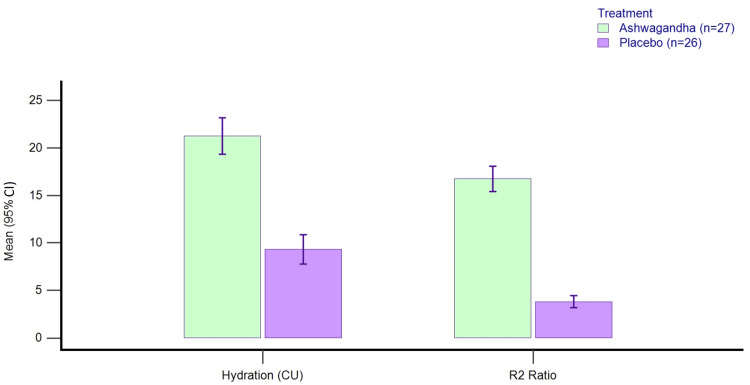
Percent change from baseline in skin hydration and sebum on day 60 in PP dataset. PP: per-protocol; R2 ratio: skin elasticity using Cutometer®; CI: confidence interval

Greater percent improvements were seen with AG as compared to PL for skin hydration (20.66% with AG and 9.5% with PL) and elasticity was assessed by the R2 ratio (16.34% with AG and 3.73% with PL) (Figure 4).

SF-12

Table 5 presents the scores of baseline and change from baseline data for SF-12 scores in the per-protocol dataset. There is a greater reduction in PCS-12, MCS-12, and total SF-12 scores with AG as compared to placebo (p<0.0001) from baseline to day 60 in the PP dataset.

**Table 5:** SF-12 (quality of life) scores in PP dataset (n=53). *Post hoc independent sample t-test (between-group comparisons). P value <0.05 for repeat measures ANOVA. QoL: quality of life; PCS-12: physical domain of SF-12 QoL; MCS-12: mental domain of SF-12 QoL; SF-12: Short Form Health Survey-12; ANOVA: analysis of variance; SD: standard deviation; CI: confidence interval; PP: per-protocol

Parameter	Treatment	N	Baseline	Change from baseline on day 60		Between-group comparisons		
			Mean (SD)	Mean (SD)	Mean difference (95% CI)	Test*	p-Value	Effect size - Cohen's “d” (95% CI)
QoL scores								
PCS-12 score	Ashwagandha	27	11.85 (2.01)	5.30 (1.79)	1.99 (1.06 to 2.91)	4.320	<0.0001	1.187 (0.596 to 1.767)
	Placebo	26	11.69 (2.13)	3.31 (1.54)				
MCS-12 score	Ashwagandha	27	17.44 (1.15)	7.30 (1.35)	2.64 (1.89 to 3.40)	7.027	<0.0001	1.931 (1.269 to 2.580)
	Placebo	26	17.08 (1.49)	4.65 (1.38)				
SF-12 total score	Ashwagandha	27	29.30 (2.61)	12.59 (2.65)	4.63 (3.22 to 6.04)	6.611	<0.0001	1.817 (1.167 to 2.453)
	Placebo	26	28.77 (2.97)	7.96 (2.44)				

**Figure 5:**
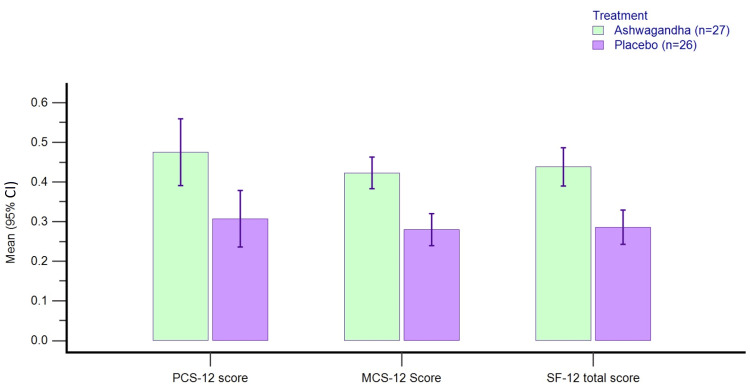
Percent change from baseline in SF-12 QoL scores on day 60 in PP dataset. QoL: quality of life; PCS-12: physical domain of SF-12 QoL; MCS-12: mental domain of SF-12 QoL; PP: per-protocol; CI: confidence interval

The percent reduction in PCS-12 scores was -46.1% with AG and -31.6% with PL, the percent reduction in MCS-12 scores was -41.8% with AG and -28.0% with PL, whereas the percent reduction in SF-12 total scores was -43.0% with AG and -28.9% with PL (Figure 5).

Compliance and adverse events

The two groups were similar with respect to the adverse events reported (p=0.718; chi-square test). Four patients from AG and five from PL reported adverse events (local irritation, erythema, and swelling) during the study period. Two (7.14%) patients from both groups reported local swelling, whereas two (7.14%) from AG and one (3.57%) patient from PL reported erythema. Local irritation was reported by two (7.14%) patients in the PL group. All events were of mild severity and no intervention was required. All events were resolved without any intervention and patients continued application of study treatments till the end of the study.

## Discussion

Facial wrinkles are important in photoaged skin, and they are caused due to structural flaws in the collagenous extracellular matrix [25]. Although wrinkle formation can happen because of free radicals on DNA, there could be reduced collagen synthesis and increased breakdown by metalloproteases. Fine wrinkles start appearing by the age of 30s, while deep wrinkles appear late in the 50s. UV rays cause histologic changes, such as damage to collagen fibers, abnormal increase in elastic fibers, and glycosaminoglycan spot pigmentation [26]. This causes the appearance of coarse wrinkles, roughness, spotted pigmentation, and skin thickening.

There is an increase in the use of plant-derived ingredients, such as Ashwagandha, saffron, l-theanine, and tocopherol for their anti-inflammatory and anti-oxidant properties to help reduce the effect of chronic stress on the skin, overall health, and quality of life [27]. Natural plant extracts contain secondary metabolites, which can inhibit, interrupt, or repair damage caused by UV exposure [28].

In this double-blind, placebo-controlled study, we evaluated the efficacy of a 60-day treatment with topical application of a lotion containing Ashwagandha root extract (8%) on the facial skin in 53 adults (age range: 18-40 years, photoaged men and women). The double-blind study design ensured blinded assessments to rule out any observer bias (global physicians’ assessment) and bias in the self-reported SF-12 quality of life survey.

Ashwagandha (*Withania somnifera*) is widely used as an adaptogen in Ayurveda and in traditional medicine [27]. Adaptogens help to cope with stressors and prevent potential physiological consequences related to stress [29]. Several studies report the efficacy and safety of Ashwagandha in conditions associated with chronic stress, anxiety, and insomnia [30]. Ashwagandha extract is reported to have depigmenting activity which may be useful for its topical action [31]. The root is the most important part of the Ashwagandha plant used in Ayurvedic preparations, and metabolomic studies revealed that metabolite profiles of Ashwagandha depended on the developmental stage of the plant, season of collection, geographical location, and part of the plant [32]. The extract used in the skin formulation for our study was prepared from a controlled plantation in India, where measures are taken to maintain the uniformity of cultivation and ensure to prevent any contamination.

The global physician assessment of skin included assessment of skin wrinkles, pores, hydration/moisture, skin brightness/tone, and pigmentation on a 6‐point Likert scale. We observed greater improvement in total score and scores for skin wrinkles and other parameters with topical application of Ashwagandha lotion (p<0.05). We assessed the skin parameters objectively using Cutometer® which is a widely accepted tool for the assessment of skin condition. We observed a greater improvement in transepidermal water loss (TEWL), skin hydration (moisture), and skin elasticity (R2 ratio) with Ashwagandha (AG) compared to placebo (p<0.0001). However, we did not observe any notable change in the melanin index with AG (-2.82%) or PL (-1.78%). There was a greater reduction (-15.12%) in TEWL with Ashwagandha after 60 days of topical application. Although there are no studies with Ashwagandha topical application, similar improvements in TEWL are reported with the use of other therapies based on plant preparations [2,33]. There was an increase in skin hydration (20.66%) and skin elasticity assessed by the R2 ratio (16.34%) with Ashwagandha. Withanolides present in the Ashwagandha extract are reported to have anti-inflammatory effects, which may be due to the suppression of mitogen‑activated protein kinase pathway, NF‑κB pathway, and cytokine expression modulation [34]. These results suggest that Ashwagandha root extract can potentially protect against skin inflammation. Topical application of fatty acids extracted from Ashwagandha seeds significantly reduced the inflammation-induced edema and repaired the psoriatic lesions and histopathological scores in 12-O tetradecanoyl phorbol 13-acetate (TPA)-induced psoriatic mouse model [35]. There was inhibition of pro-inflammatory cytokines release and reactive nitrogen species (RNS) in lipopolysaccharide (LPS)-stimulated RAW264.7 cells.

We also assessed the effect of topical therapy on quality of life using the Short Form Health Survey-12 (SF-12), which is a validated questionnaire and widely accepted in research applications [36]. Other research in skin improvement has used other quality of life scales like Profile of Mood States (POMS), and Pittsburgh Sleep Quality Index (PSQI) Survey) [27]. The self-reported SF-12 survey evaluates the physical and mental state of the responders. We observed a greater reduction in PCS-12, MCS-12, and total SF-12 scores with AG as compared to placebo (p<0.0001). Skin being a cosmetic organ, skin quality can have a direct impact on the quality of life in both children and adults [37,38]. Ashwagandha being an adaptogen could improve the quality of life through mechanisms other than improvement in skin quality [15].

This study had several limitations. Our study population was limited by a small sample size of 53 evaluable participants and participants were less than 40 years of age and wanted to improve their skin condition for cosmetic purposes. Additionally, the study duration was limited to 60 days, suggesting a need for longer follow-up duration. Our study did not involve oral therapy which could have potential additional benefits. Future studies should include an expanded population with a wider age range, standard therapies as comparators, and larger sample size that may include different skin types.

## Conclusions

This study assessed the efficacy and safety of topical application of Ashwagandha lotion on the improvement of skin parameters in photoaged facial skin of healthy subjects. Ashwagandha improved the total scores for physicians' assessment of five signs/symptoms (skin wrinkles, pores, hydration/moisture, skin brightness/tone, and pigmentation) on facial skin. There was a reduction in transepidermal water loss (TEWL) and an increase in skin hydration and elasticity assessed by Cutometer. The melanin index was not affected by Ashwagandha application. Topical application of lotion containing Ashwagandha standardized root extract improves the skin condition and quality of life in photoaged healthy individuals. However, further studies with different skin types and standard comparators are warranted to substantiate these claims of benefit.
